# Regression of metastatic colon tumour from primary adenocarcinoma of the lung due to fistulisation to the bowel lumen

**DOI:** 10.3332/ecancer.2014.412

**Published:** 2014-03-20

**Authors:** Masaya Iwamuro, Yoshio Miyabe, Kai Hanae, Kawai Yoshinari, Takata Katsuyoshi, Toshi Murakami, Mifune Hirofumi, Kazuhide Yamamoto

**Affiliations:** 1 Department of Gastroenterology, Onomichi Municipal Hospital, Onomichi 722-8503, Japan; 2 Department of Gastroenterology and Hepatology, Okayama University Graduate School of Medicine, Dentistry, and Pharmaceutical Sciences, Okayama 700-8558, Japan; 3 Department of Pathology, Okayama University Graduate School of Medicine, Dentistry, and Pharmaceutical Sciences, Okayama 700-8558, Japan; 4 Department of Respiratory Medicine, Onomichi Municipal Hospital, Onomichi 722-8503, Japan; 5 Department of Radiology, Onomichi Municipal Hospital, Onomichi 722-8503, Japan

**Keywords:** colonic metastasis, lung cancer, pulmonary adenocarcinoma, tumour-bowel fistula

## Abstract

An 80-year-old Japanese male was diagnosed with pulmonary adenocarcinoma. The patient exhibited extensive extra pulmonary involvement in the bone, adrenal gland, abdominal lymph nodes, and sigmoid colon. A single course of chemotherapy with carboplatin and pemetrexed was administered as the first-line treatment. Subsequently, the patient received pemetrexed monotherapy. Two months after the diagnosis, rapid regression of the metastatic tumour in the sigmoid colon was observed. Based on the findings of CT scanning and colonoscopic examination, tumour-bowel fistulisation was considered to be a cause of the rapid regression. This case report illustrates a tumour-bowel fistula of a colonic metastatic tumour in a patient with lung cancer. Radiographic and endoscopic features of the rare manifestation are presented.

## Introduction

Distant metastases are frequently encountered manifestations in patients with lung cancer. They can be present at the initial diagnosis or can occur in the treatment period. The bones, central nervous system, adrenal glands, and liver are representative organs involved in distant metastases [1–3]. On the other hand, the involvement of the intestinal tract is uncommon. Patients with intestinal metastasis may manifest a wide range of symptoms, including asymptomatic abdominal mass, pain, intestinal bleeding, ileus, and perforation of the intestinal tract [4]. Fistulisation of metastatic tumours to the intestinal lumen may also occur, but it is rare.

We recently treated a patient with lung cancer who had extensive extra pulmonary involvement in the bone, adrenal grand, intra-abdominal lymph nodes, and sigmoid colon. Rapid regression of the metastatic tumour in the sigmoid colon was observed, and tumour-bowel fistulisation was considered to be a cause. Herein, we report the case and illustrate its radiographic and endoscopic features.

## Clinical case

An 80-year-old Japanese male underwent a chest x-ray examination at his family clinic due to the presence of dull pain around his shoulder. The patient was diagnosed with a lung tumour, and was referred to Onomichi Municipal Hospital for further investigation and treatment. He had a previous history of myocardial infarction and hypertension. He had a heavy smoking history of 80 cigarettes per day for 50 years. The patient had no history of pulmonary or gastrointestinal disease. Physical examination revealed no abnormalities. Laboratory studies revealed significantly elevated levels of carcino embryonic antigen (CEA; 466.3 ng/mL) and sialyl stage-specific embryonic antigen-1 (SLX; 175.8 U/mL). Cytokeratin 19 fragment (CYFRA) concentration was also elevated (7.6 ng/mL). The levels of squamous cell carcinoma (SCC)-related antigen and gastrin discharge peptide precursor (ProGRP) were within the normal ranges.

**Figure 1.**
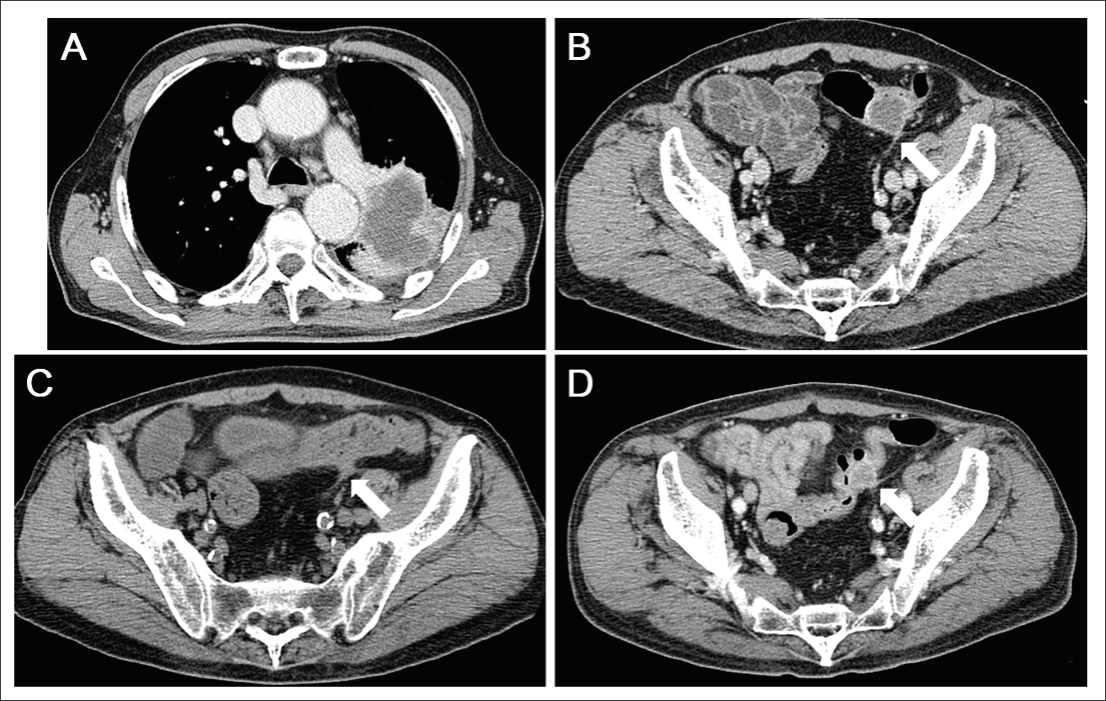
CT scanning images. (a) Lung cancer in the left-upper lobe measuring 7.8 cm in diameter. The tumour invaded both the pleura and the outer layer of the descending aorta. Central necrosis was shown. (b) A metastatic tumour in the sigmoid colon. (c) During chemotherapy, the tumour in the sigmoid colon decreased in size, whereas the primary lung tumour and other metastatic tumours were unchanged. (d) Four months after the diagnosis of lung cancer, the sigmoid colon tumour was enlarged again.

**Figure 2.**
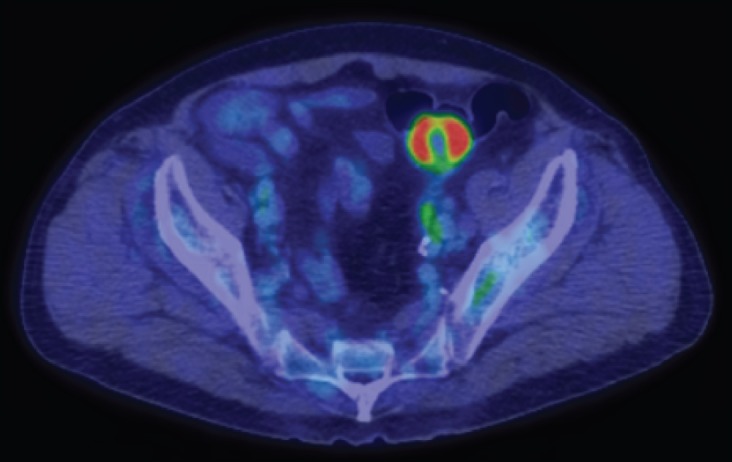
Positron emission tomography scanning showed tracer uptake in the sigmoid colon tumour. Central necrosis was observed.

**Figure 3.**
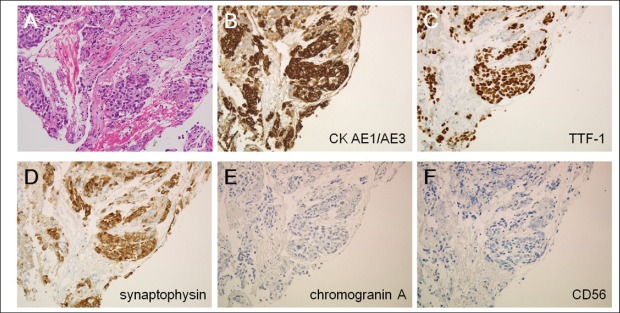
Histological examinations of the lung tumour. (a) Atypical cells with a high nuclear-cytoplasmic ratio were observed in the biopsied specimen (haematoxylin and eosin staining). (b) These cells were positive for cytokeratin AE1/AE3, (c) thyroid transcription factor-1, and (d) synaptophysin, but were negative for (e) chromogranin A and (f) CD56 (original magnifications: x20). These results were consistent with adenocarcinoma of the lung.

CT scanning revealed a tumour in the left-upper lobe of the lung of 7.8 cm in diameter (Figure 1a). The tumour invaded both the pleura and the outer layer of the descending aorta. The peripheral part of the tumour was enhanced with the contrast medium, whereas the inner-part lacked enhancement, suggesting central necrosis. CT scanning showed multiple tumours in the thoracic vertebra, lumbar vertebra, adrenal gland, and sigmoid colon. The colonic tumour was 2.8 cm in diameter. The surface layer of the tumour showed enhancement with contrast media, but most of the tumour itself lacked enhancement (Figure 1b). These features suggested central necrosis and were quite similar to those of the pulmonary tumour. Swelling of the para-aortic lymph nodes was also seen. Positron emission tomography scanning showed tracer uptake in the tumours and para-aortic lymph nodes. Because tracer accumulation was seen in the peripheral part of the sigmoid colon tumour (Figure 2), a tumour with central necrosis was highly suspected. A biopsy of the lung tumour was performed by bronchoscopy, and the specimen revealed adenocarcinoma of the lung (Figure 3), with a polymerase chain reaction-invader assay showing a negative *EGFR* mutation status. The *EML4-ALK* fusion gene was also negative.

**Figure 4.**
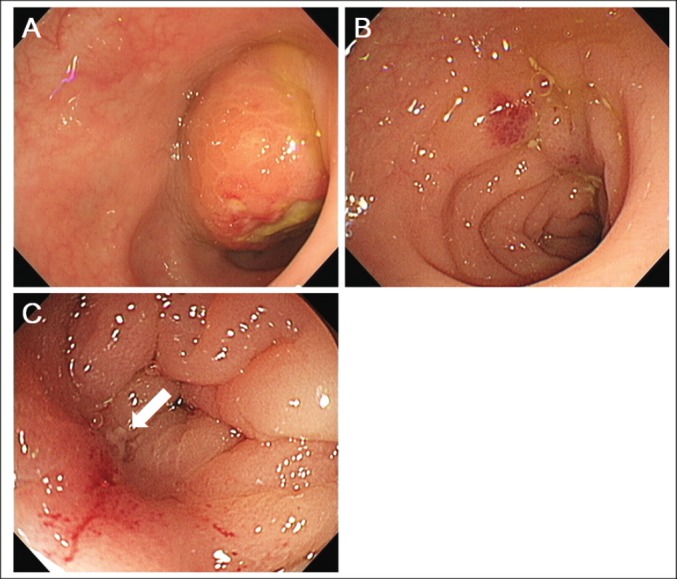
Colonoscopic examination images. (a) A submucosal tumour with ulceration was seen in the sigmoid colon. (b) Two months after the diagnosis, the tumour had disappeared and a longitudinal ulcer was seen. (c) Close-up observation revealed pus-like discharge from the ulcer, suggesting tumour-bowel fistulisation.

**Figure 5.**
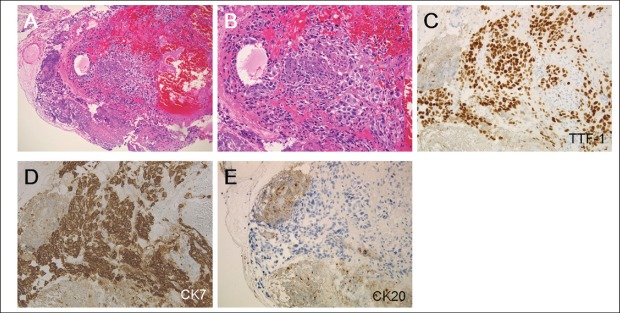
Histological examinations of the sigmoid colon tumour. (a and b) Atypical cells with a high nuclear-cytoplasmic ratio and fine nuclear chromatins (haematoxylin and eosin staining, a: x10, b: x20). (c) The cells were positive for thyroid transcription factor-1 and (d) cytokeratin 7, but were negative for (e) cytokeratin 20 (c–e: x20). Consequently, a diagnosis of metastasis of pulmonary adenocarcinoma to the sigmoid colon was made.

Colonoscopic examination revealed a submucosal tumour in the sigmoid colon, which had ulceration on its top (Figure 4a). Biopsy samples taken from the colonic tumour showed a proliferation of atypical cells with a high nuclear-cytoplasmic ratio and fine nuclear chromatins (Figure 5). The morphology of these cells was similar to that of the pulmonary cancer cells. In immunohistochemical analysis, the neoplastic cells were positive for cytokeratin 7 and thyroid transcription factor-1 but negative for cytokeratin 20. Consequently, a diagnosis of lung adenocarcinoma with multiple metastases to the bone, adrenal gland, and sigmoid colon was made. The patient’s clinical stage was T4N1M1b.

A single course of chemotherapy with carboplatin and pemetrexed was administered as the first-line treatment. Subsequently, the patient received pemetrexed monotherapy. Two months after the diagnosis, the patient underwent CT scanning because he exhibited a high fever. Inflammation around the kidney was detected by CT scanning, and pyuria was observed. Consequently, pyelonephritis was suspected as the cause of his fever. In addition, CT scanning showed the primary lung tumour and metastatic tumours to be unchanged, except that the sigmoid colon tumour had obviously regressed to 0.8 cm in diameter (Figure 1c). The necrotic area seen in the colonic tumour had completely disappeared. Colonoscopic examination showed that a submucosal tumour had disappeared, and a longitudinal ulcer was seen instead (Figure 4b). Close-up observation revealed a pus-like discharge from the ulcer (Figure 4c), suggesting tumour regression following fistulisation into the bowel lumen. The patient died due to severe pneumonia four months after the diagnosis of lung cancer. CT scanning performed a day before his death revealed that the primary lung tumour and metastatic tumours remained unchanged in size, but the sigmoid colon tumour was enlarged again to 1.8 cm in diameter (Figure 1d). The surface showed strong enhancement and the central part was slightly enhanced with contrast media, which suggested a proliferation of metastatic tumour cells.

## Discussion

Approximately, 50% of patients with lung cancer have distant metastasis at the initial diagnosis [5, 6]. Previous reports of autopsy studies have found the rate of gastrointestinal metastasis from primary lung cancer to be unexpectedly high, up to 14% [7–11]. In the clinical setting, however, involvement of the gastrointestinal tract is less common compared with other organs, such as the bone, brain, adrenal glands, and liver. This discrepancy between clinical and postmortem findings can be explained by the observation that metastatic lesions in the gastrointestinal tract rarely give rise to symptoms or complications [7, 12]. For example, it has been reported that only 0.4–0.5% of gastrointestinal metastases from lung cancer lead to the occurrence of symptoms [7, 13, 14].

Within the gastrointestinal tract, the small intestine is the most frequently affected area, whereas colonic metastasis from lung cancer is extremely uncommon. Rossi *et al* [15], in a summary of results for 18 lung cancer patients with gastrointestinal metastasis, found that the metastasis most commonly involved the small intestine (*N* = 12), with fewer cases involving the stomach (*N* = 4) or large intestine (*N* = 2). Sakai *et al* noted that only 12 lung cancer cases with metastases to the large intestine have been reported in published case reports as of 2012 [5, 6, 16–24]. In our literature review, we found four additional cases that had been recently published [1, 25, 26]. In the 17 documented cases, including these 16 previous cases and our case, the median age was 63 years (43–80 years), and there is a clear male predominance (*N* = 15, 88.2%). The pathological subtypes of the reported cases were SCC (*N* = 13), adenocarcinoma (*N* = 2) [25], small cell carcinoma (*N* = 1), and large cell carcinoma (*N* = 1). The sigmoid colon is the most frequently metastasised site (*N* = 11), followed by the caecum (*N* = 3), ascending colon (*N* = 1), transverse colon (*N* = 1), and descending colon (*N* = 1). Therefore, a representative example of metastasis from lung cancer to the large intestine is a male patient with pulmonary SCC metastasising to the sigmoid colon.

Tumour-bowel fistula is one of the potential complications of malignancies in the abdominopelvic cavity [27, 28]. The development of a fistula may be associated with spontaneous tumour growth or with cancer treatments, such as chemotherapy and/or radiotherapy [29]. Although a variety of symptoms including abdominal pain, nausea, vomiting, diarrhoea, bleeding, and fever have been reported as manifestations of tumour-bowel fistula, such fistulae can be asymptomatic. In the present case, fistulisation of the sigmoid colon tumour was coincidentally suspected as a result of CT scanning, which was performed to localise the source of the fever. Subsequently, the fistulisation was confirmed by colonoscopy. Though pyelonephritis was considered to be the main cause of his fever, the tumour-bowel fistula might have been responsible for the fever to some extent. Generally, a tumour-bowel fistula can cause either a decrease in the size of the tumour by evacuating the content into the bowel lumen, as described in this case, or an increase in the size of the tumour by drawing intestinal contents into the tumour [27, 28].

The management of tumour-bowel fistula has not been established to date. Possible treatments for symptomatic patients include discontinuation of the preceding chemotherapeutic agents, administration of antibiotics, surgical resection of the tumour and fistula, and percutaneous drainage of the tumour abscess [27]. We conclude that patients with tumour-bowel fistula must be treated on a case-by-case basis, because such patients may have severe comorbidities and widespread metastases, or may be in a poor clinical status.

## Conclusion

We experienced a rare case of primary lung cancer with metastasis to the sigmoid colon. The metastatic tumour rapidly regressed due to fistulisation into the bowel wall. Although such manifestations are infrequent, tumour-bowel fistula has to be suspected when a tumour close to the intestinal tract rapidly decreases in size.

## References

[1] Azevedo CR (2010). Synchronous thyroid and colon metastases from epidermoid carcinoma of the lung: case report. Sao Paulo Med J.

[2] Quint LE (1996). Distribution of distant metastases from newly diagnosed non-small cell lung cancer. Ann Thorac Surg.

[3] Hillers TK, Sauve MD, Guyatt GH (1994). Analysis of published studies on the detection of extrathoracic metastases in patients presumed to have operable non-small cell lung cancer. Thorax.

[4] Uygun K (2006). Colonic metastasis from carcinoma of the breast that mimics a primary intestinal cancer. Yonsei Med J.

[5] Sakai H (2012). Primary lung cancer presenting with metastasis to the colon: a case report. World J Surg Oncol.

[6] Yang CJ (2006). Gastro-intestinal metastasis of primary lung carcinoma: clinical presentations and outcome. Lung Cancer.

[7] Goh BK (2007). Laparotomy for acute complications of gastrointestinal metastases from lung cancer: is it a worthwhile or futile effort?. Surg Today.

[8] McNeill PM, Wagman LD, Neifeld JP (1987). Small bowel metastases from primary carcinoma of the lung. Cancer.

[9] Antler AS (1982). Gastrointestinal metastases form malignant tumors of the lung. Cancer.

[10] Burbige EJ, Radigan JJ, Belber JP (1980). Metastatic lung carcinoma involving the gastrointestinal tract. Am J Gastroenterol.

[11] Stenbygaard LE, Sørensen JB (1999). Small bowel metastases in non-small cell lung cancer. Lung Cancer.

[12] Goh BK (2006). Upper gastrointestinal bleed secondary to duodenal metastasis: a rare complication of primary lung cancer. J Gastroenterol Hepatol.

[13] Garwood RA (2005). A case and review of bowel perforation secondary to metastatic lung cancer. Am Surg.

[14] Berger A (1999). Small bowel metastases from primary carcinoma of the lung: clinical findings and outcome. Am J Gastroenterol.

[15] Rossi G (2007). Primary lung cancer presenting with gastrointestinal tract involvement: clinicopathologic and immunohistochemical features in a series of 18 consecutive cases. J Thorac Oncol.

[16] Gitt SM (1992). Bowel perforation due to metastatic lung cancer. J Surg Oncol.

[17] Hirasaki S (2008). Asymptomatic colonic metastases from primary squamous cell carcinoma of the lung with a positive fecal occult blood test. World J Gastroenterol.

[18] Stinchcombe TE (2006). Lung cancer presenting with a solitary colon metastasis detected on positron emission tomography scan. J Clin Oncol.

[19] Habeşoğlu MA (2005). A case of bronchogenic carcinoma presenting with acute abdomen. Tuberk Toraks.

[20] Carroll D, Rajesh PB (2001). Colonic metastases from primary squamous cell carcinoma of the lung. Eur J Cardiothorac Surg.

[21] Bastos I (1998). Colonic metastasis of a lung carcinoma with ileocolic fistula. J Clin Gastroenterol.

[22] Gateley CA, Lewis WG, Sturdy DE (1993). Massive lower gastrointestinal haemorrhage secondary to metastatic squamous cell carcinoma of the lung. Br J Clin Pract.

[23] Brown KL (1980). Rare metastasis of primary bronchogenic carcinoma to sigmoid colon: report of a case. Dis Colon Rectum.

[24] Smith HJ, Vlasak MG (1978). Metastasis to the colon from bronchogenic carcinoma. Gastrointest Radiol.

[25] Pezzuto A (2013). Metastasis to the colon from lung cancer presenting with severe hyponatremia and dyspnea in a young male: a case report and review of the literature. Oncol Lett.

[26] Mehta RS (2013). Lung cancer with gastrointestinal metastasis - review of theories of metastasis with three rare case descriptions. Cancer Microenviron.

[27] Tirumani SH (2013). Tumor-bowel fistula: what radiologists should know. Abdom Imaging.

[28] Tirumani SH (2014). Multidetector-row CT of tumour-bowel fistula: experience at a tertiary cancer centre. Clin Radiol.

[29] Chow H (2011). Tumor fistulization associated with targeted therapy: computed tomographic findings and clinical consequences. J Comput Assist Tomogr.

